# Social Support Among Women With Potential Essure-Related Complaints: Analysis of Facebook Group Content

**DOI:** 10.2196/32592

**Published:** 2023-08-03

**Authors:** Daniëlle van Gastel, Marjolijn L Antheunis, Kim Tenfelde, Daniëlle L van de Graaf, Marieke Geerts, Theodoor E Nieboer, Marlies Y Bongers

**Affiliations:** 1 Research School GROW University Maastricht Maastricht Netherlands; 2 Tilburg School of Humanities and Digital Sciences Tilburg University Tilburg Netherlands; 3 Radboud University Medical Center Nijmegen Netherlands

**Keywords:** Essure, social support, Facebook, sterilization, patient online communities, social media, social networks

## Abstract

**Background:**

Social support groups are an important resource for people to cope with problems. Previous studies have reported the different types of support in these groups, but little is known about the type of reactions that sharing of personal experiences induce among members. It is important to know how and to what extent members of support groups influence each other regarding the consumption of medical care. We researched this in a web-based Facebook group of women sterilized with Essure. Essure was a device intended for permanent contraception. From 2015 onward, women treated with Essure for tubal occlusion raised safety concerns and numerous complaints.

**Objective:**

This study aimed to evaluate the use of social support in a Facebook community named “Essure problemen Nederland” (EPN; in English, “Essure problems in the Netherlands”).

**Methods:**

All posts in the closed Facebook group EPN between March 8 and May 8, 2018, were included. In total, 3491 Facebook posts were analyzed using a modified version of the Social Support Behavior Codes framework created by Cutrona and Suhr in 1992. Posts were abstracted and aggregated into a database. Two investigators evaluated the posts, developed a modified version of the Social Support Behavior Codes framework, and applied the codes to the collected data.

**Results:**

We found that 92% of messages contained a form of social support. In 68.8% of posts, social support was provided, and in 31.2% of posts, social support was received. Informational and emotional support was the most frequently used form of provided social support (40.6% and 55.5%, respectively). The same distribution was seen with received social support: informational support in 81.5% and emotional support in 17.4% of cases. Our analysis showed a strong correlation between providing or receiving social support and the main form of social support (*P*<.001). In a total of only 74 (2.2%) cases, women advised each other to seek medical care.

**Conclusions:**

The main purpose of women in the EPN Facebook group was to provide and receive informational or emotional support or both.

## Introduction

Social support groups are an important resource for people to cope with a serious illness, as they provide support for those in need [1]. In previous decades, an increasing number of people participate in web-based social support groups. Web-based groups are not restricted by the temporal, geographical, and spatial limitations typically associated with face-to-face groups [2]. Although previous studies have reported the different types of support in these groups, little is known about the type of reactions that sharing of personal experiences induce among members [3,4]. It is important to know how and to what extent members of support groups influence each other regarding the consumption of medical care. In order to evaluate the sequence of seeking and providing of social support, a content analysis of actual social support messages on social support platforms is needed.

In this study, we focused on a web-based social support group on problems with Essure. Essure (Bayer AG) was a device intended for permanent contraception. The advantages of this form of sterilization were its minimally invasive character and the possibility to perform the tubal occlusion in an outpatient clinic without the need for general anesthesia. From 2015 onward, women treated with Essure for tubal occlusion raised safety concerns. A large number of adverse events were reported to the Food and Drug Administration [5]. The most frequently mentioned and important complaints were abdominal or pelvic pain (or both), fatigue, and abnormal vaginal bleeding. Since March 2016, an increasing number of Dutch women requested surgical removal after the broadcast of a TV program in the Netherlands. Earlier, in March 2015, Dutch women established a private Facebook social support community, “Essure problemen Nederland” (EPN; in English, “Essure problems in the Netherlands”). Currently, more than 3900 women are united in this Facebook social support community. These women share their own personal stories and information about the device, the gynecologists performing Essure removal surgery, the hospitals, and the surgery.

The primary aim of this study is to evaluate the sequence of seeking and providing social support on the EPN social support page on Facebook. More specifically, we aim to investigate how types of social support in original (first) posts relate to types of social support in the reactions to these posts. Our second aim is to evaluate the specific content of the messages and whether women sterilized with Essure urged each other to seek more or less medical care as a result of the social support.

## Methods

### Sample

In May 2018, a total of 3491 Facebook posts were automatically retrieved with a script from the EPN Facebook social support group. The EPN group was created by 2 women who used Essure to meet the informational and emotional needs of women treated with Essure devices and particularly those who experienced usage-related issues. EPN is a closed group, which means that one has to be accepted by the administrator (or administrators) to become a member in order to be able to read and post messages. During the data collection process in May 2018, the group had a little over 3800 members, mainly consisting of Dutch women sterilized using Essure devices. All posts retrieved for this study were posted between March 8 and May 8, 2018. Introductory and pinned posts by the EPN Facebook moderators were not included in the data.

### Ethics Approval

The research ethics committee of the Radboud University Medical Centre, Nijmegen declared that this study did not fall within the remit of the Medical Research Involving Human Subjects Act. Ethical approval was also obtained from institutional review board of the Radboud University Medical Centre, Nijmegen (2018-4143) and from the research ethics committee of Tilburg School of Humanities and Digital Sciences (REC # 2018/26).

### Procedure

The administrators of the EPN Facebook were approached by a study researcher (TEN). The administrators approved the use of messages posted on the EPN Facebook group for the study and announced in a post on the page the purpose of the study, information about the data collection process, and how privacy of the participants would be ensured. Members could refuse their posts and reactions to be included in the database. This was done based on the opt-out method in which the member had to email the researcher with her refusion to participate. The members were provided enough time to read the message and email the researcher. Four EPN Facebook members indicated that they did not want to have their messages be included in the study; hence, their messages were deleted from the data set. For the other participants, names were replaced by “[NAME]” to preserve their privacy. After anonymizing the data set, the coding of the messages was carried out by 2 communication and information students from Tilburg University.

### Measures

The chosen unit for coding was based on the content of the whole post. All posts that included social support (N=3073) were coded. These messages were analyzed using a codebook specifically designed for the purpose of this study. The first part of the codebook was about elementary information regarding the post, containing questions about the origin of the message (original posts or replies), the format, and the provision or receipt of social support in the post.

The second part of the codebook was based on the Social Support Behavior Code (SSBC) framework developed by Cutrona and Suhr [6] in 1992. The original scale was intended to code social support present in real-life conversations. For this study, the SSBC was adapted to fit web-based social support, as it has been previously successfully used as a coding system for analyzing web-based social support bulletin messages. This taxonomy was designed to assess the frequency of occurrence of support-intended communication behaviors that fall within six main categories: (1) informational support, (2) esteem support, (3) network support, (4) emotional support, (5) tangible assistance, and (6) other. The first 5 main categories had several subthemes. For example, subthemes under category 1—informational support—include advice, referral to experts, teaching, announcement, situation appraisal, and personal experience. The subtheme “listening” was removed, as this is not possible digitally. In addition, the subcategory *psychical affection* was replaced by digital affection [7,8]. Table 1 shows all the 26 subthemes belonging to the 5 main categories. The coders could code 2 main categories per post, and then 1 subtheme per main category.

The third part of the codebook was not based on the type of social support but rather the content of the message. The list of categories was preselected and based on the clinical experiences of the researchers and gynecologists. This was intended to evaluate the kind of information and advice people are disseminating in the Facebook group. The codebook described specific topics that were monitored, such as advice about recovery after surgery, encouragement for visiting a health care provider, or positive information about a doctor on the list. The owners of this community developed a list of top doctors or gynecologists for removal of the Essure devices, some listed as even more specialized to remove lost fragments and small parts of no more than 1-2 mm of the device that sometimes tear off during the removal of the Essure devices. Contrary to the manner of coding for the SSBC framework, this information was allowed to be coded on a more sentence level rather than from the content of the whole post, due to the information needed. In every message, the coders could choose a maximum of 3 contents as shown in Tables 2 and 3.

**Table 1:** Posts in each social support category.

Social support category			Provided, n (%)		Received, n (%)		Total, n (%)
**Information**							1640 (53.4)
	Advice	394 (18.7)		206 (21.5)		600 (19.5)	
	Referral to experts	5 (0.2)		0 (0)		5 (0.2)	
	Situation appraisal	66 (3.1)		1 (0.1)		67 (2.2)	
	Teaching	70 (3.3)		0 (0)		70 (2.3)	
	Announcement	2 (0.1)		1 (0.1)		3 (0.1)	
	Personal experience	322 (15.2)		574 (59.9)		896 (29.2)	
**Network**							27 (0.9)
	Access	18 (0.9)		0 (0)		18 (0.6)	
	Presence	6 (0.3)		0 (0)		6 (0.2)	
	Companionship	3 (0.1)		0 (0)		3 (0.2)	
	Sharing stories	0 (0)		0 (0)		0 (0)	
**Esteem**							48 (1.6)
	Compliment	15 (0.7)		1 (0.1)		16 (0.5)	
	Validation	18 (0.9)		8 (0.8)		26 (0.8)	
	Relief of blame	3 (0.1)		0 (0)		3 (0.1)	
	Feedback/opinion	2 (0.1)		1 (0.1)		3 (0.1)	
**Emotional**							1340 (43.6)
	Relation	0 (0)		0 (0)		0 (0)	
	Virtual affection	2 (0.1)		1 (0.1)		3 (0.1)	
	Sympathy	202 (9.6)		15 (1.6)		217 (7.1)	
	Empathy	144 (6.8)		34 (3.5)		178 (5.8)	
	Encouragement	627 (29.7)		3 (0.3)		631 (20.5)	
	Prayer	0 (0)		0 (0)		0 (0)	
	Consoling	1 (0)		0 (0)		1 (0)	
	Expression of gratitude	16 (0.8)		114 (11.9)		130 (4.2)	
	Congratulation	180 (8.5)		0 (0)		180 (5.9)	
**Tangible**							16 (0.5)
	Perform task	14 (0.7)		0 (0)		14 (0.5)	
	Active participation	1 (0)		0 (0)		1 (0)	
	Willingness	1 (0)		0 (0)		1 (0)	
Others			0 (0)		0 (0)		2 (0.1)
Total			2112 (100)		959 (100)		3073 (100)

**Table 2:** Number and percentage of specific contents of the postings.

Specific content of posts	Posts, n (%)
Sharing information about the surgery and recovery	286 (8.6)
Sharing positive experiences or recommendations about a doctor from the list	107 (3.2)
Recommend undergoing surgery by a doctor from the list	50 (1.5)
Wishing good luck	752 (22.5)
Sharing information of a fragment of the device	58 (1.7)
Recommend doing something about the fragment	6 (0.2)
Asking about recognition of complaints before or after the surgery, including menses	48 (1.4)
Asking for advice regarding vitamins of nutrition	19 (0.6)
Others	2015 (60.3)

**Table 3:** Initiate a visit to a caregiver.

	Posts, n (%)
No impetus to visit or call a care provider	3268 (97.8)
Initiate a visit or call to a general practitioner	12 (0.4)
Initiate a visit or call to a gynecologist	3 (0.1)
Initiate a visit or call to a physiotherapist	3 (0.1)
Initiate a visit or call to an osteopath	6 (0.2)
Initiate a visit or call to a gynecologist who removes fragments	22 (0.7)
Initiate a visit or call to a hospital	9 (0.3)
Initiate a visit or call to a medical institution	19 (0.6)
Total	3342 (100)

### Content and Statistical Analyses

First, a content analysis of the social support messages was conducted to investigate the frequency of the various types of social support present in the EPN group. The anonymous posts were coded by 2 well-trained coders based on the codebook. We trained the coders thoroughly in multiple meetings, in which we went through the codebook in an in-depth manner. We took subsamples of posts to determine whether the codebook was detailed enough and all relevant categories were included. After these first training sessions, a subsample of 152 messages were coded as a trial and to determine whether everything was clear. After the coding of these messages, the coders discussed the remaining uncertainties and inequalities in the coding led by the main researchers. Based on this meeting, the final adjustments were made in the codebook. Next, to determine intercoder agreement, both coders coded the same 373 messages of the data set (11% of the total data set). Intercoder agreement was very high, with a percentage of 97.8%. The scores per variable were as follows: origin of the message (1.00), format of the message (1.00), social support in the message (0.99), orientation of the social support (giving or receiving; 0.97), social support category (0.90), specific content (0.93), encouragement recovery (0.99), and encouragement to seek medical health care (0.99). Subsequently both coders coded half of the remaining posts.

Statistical analysis was performed using SPSS software (version 24; IBM Corp). All analysis were performed with the 1-sample parametric chi-square test. To evaluate how types of social support in original posts relate to types of social support in the reactions, a new variable was generated.

## Results

The included posts (N=3491) in the data set consisted of 306 original posts, 3036 reactions to these original posts (with a maximum of 11 reactions per original post), and 149 picture posts (displayed as empty fields in the data set). As this study—and the codebook—focused on written text social support, these messages were deleted from the data set, yielding a data set of 3342 posts.

**Figure 1:**
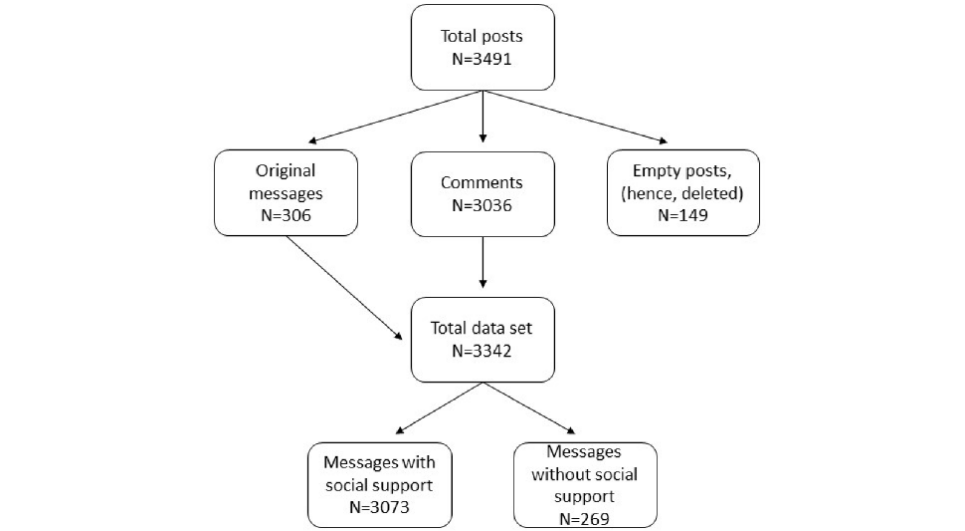
Study flowchart.

Of the 3342 posts with text, 3073 (92%) messages contained a form of social support. In 268 (8%) messages, no form of social support was present (Figure 1). Posts without social support were mostly from people getting tagged or short reactions to others, such as “yes got it.” In 2114 (68.8%) posts, social support was provided and in 959 (31.2%) posts, social support was received (Figure 1). Our analysis showed a strong correlation between providing or receiving social support and the main form of social support. Our analysis showed significant coherence (*χ*^2^_5_=449,493; *P*<.001). In the 2114 posts in which social support was provided, emotional support had the highest prevalence (n=1173). In addition, in 959 posts in which social support was received, informational support was the most frequent form of social support (n=782).

Table 1 shows the frequency of each main category of social support and 26 subcategories. Informational and emotional support were the most frequent form of provided social support (n=859, 40.6% and n=1172, 55.5%, respectively). Esteem, network, and tangible social support seem to be less frequently offered. The same distribution was seen with receiving social support: informational support in 81.6% (n=782) and emotional support in 17.4% (n=167) of cases. Informational support mainly consisted of women sharing personal experiences (n=896, 29.2%) and providing advice (n=600, 19.5%). Our analysis revealed a strong correlation between informational support and sharing personal information (*χ*^2^_88_=12,281,224; *P*<.001). One research question focused on the relationship between sharing of personal experiences in the original post and the types of social support in the responses. A new selection variable was generated, and the chi-square test showed that emotional support, with the subcategory *encouragement*, is the most common type of social support in the responses to messages with personal experiences. Coherence was significant (*χ*^2^_22_=308,566; *P*<.001).

Table 2 describes the specific contents of the Facebook messages. In 752 (22.2%) posts, women wished each other good luck or well-being. In addition, women share in 8.6% (n=286) of the posts information about their surgery or recovery (or both). Overall, 60% of cases are categorized under others, in most of these cases, the women congratulated each other on the surgery date or recognized the complaints mentioned earlier.

Table 3 shows the number of times women motivate each other to seek medical health care. As shown, in 22 posts, women urged one another to seek aid from a gynecologist who can remove retained fragments of the Essure device. In 0.6% (n=19) of the messages, they initiated a call to a medical institution. For example, one woman responded as follows: “….if you are feeling nauseous, you should contact the doctor!” In a total of 74 (2.2%) cases, women advised one another to seek medical care. In some cases, women reassured each other by recognizing symptoms, in cases where there was no incentive to visit a medical institution. One woman posted the following: “I had surgery 4 weeks ago, it went very well, but now after 4 weeks I have a relapse. My symptoms are abdominal pain, burning feeling in my vagina and bloated abdomen. Should I contact the hospital?” Another women responded as follows: “This is normal during the recovery time” and “Completely normal, I had the same symptoms, it will go better in time.”

## Discussion

### Principal Findings

In this study, we aimed to analyze the use of social support (providing and seeking) in a private Facebook group with women reporting complaints potentially related to Essure devices, and whether the social support urged users to seek more or less medical care. Our study contributes to the current research and reports that sharing personal experiences leads to showing encouragement in the responses. This study is unique because it has been conducted in a web-based social support group for women with a specific complaint about Essure sterilization devices. The majority of previous studies on web-based social support groups focused on groups for people with mental problems or in groups for people with terminal illnesses (eg, cystic fibrosis, Huntington disease, and autism spectrum disorders) [3,4,7-10]. Few studies have been conducted for nonterminal health problems. This study, therefore, complements the current research results.

This study shows the frequency of types of social support in a web-based community for women having undergone tubal occlusion with Essure devices, using the SSBC framework of Cutrona and Suhr [6]. We found that more than 90% of messages regarded informational and emotional support, both in providing and receiving support. Furthermore, we found that emotional support occurred most frequently within a message in which social support was provided and informational support occurred most frequently in messages where social support was received. In addition, we looked at the specific content of the messages and found that in 0.6% (n=19) and 0.7% of cases, women advised each other to seek aid from a medical doctor.

Our results are in line with those of previous studies that reported that informational support is the most common type of support in social support communities, followed by emotional support (53.4% and 43.6%, respectively) [3,8,11,12]. Support posts relating to network and esteem were exchanged at similar frequencies as those reported in other studies, while tangible support was the least frequent form among the 5 types of social support. This can be attributed to the web-based nature of the group. Group members live across the country, reducing their capability for exchanging anything tangible or responding to offers of physical assistance.

Sharing personal information is an important motivation for people to use web-based social support groups [13]. Therefore, we expected that most messages in the web-based support group would consist of sharing personal information as is confirmed by our results. We found that informational support combined with sharing of personal experiences occurred the most and is the most important activity within a web-based social support group. This result is consistent with that of content analysis of bulletin boards for chronic conditions [8]. In addition, it mirrors the findings from the literature on face-to-face social support groups.

Another purpose of this study was to evaluate the social support used and to investigate how types of social support in original messages relate to types of social support in the reactions to these messages. Therefore, we investigated how sharing personal information in original messages relates to the content of the responses within this web-based support group. Hodges et al [14] and other studies [2,15] suggested that empathy was particularly observed in the responses to informational support. We found that emotional support with the subcategory *encouragement* was the most frequent response to posts in which personal information was shared. This is comparable to the findings of Coulson et al [8], in that providing emotional support was an important aspect of this group. The subcategory *encouragement* consists of acknowledging others’ feelings and validating them by reiterating their own similar views and experiences.

A final goal of this study was to view the specific content of the messages in the group to determine the individuals’ motivation to contact a health care professional. We expected, based on clinical experience, that women advise each other to seek medical care to remove the devices. However, in most situations, women reassure each other by recognizing complaints instead of advising to seek medical care. The fact that women in our study reassured one another instead of encouraging one another to seek medical care, was remarkable in the case of Essure, however, without delay of necessary medical care to our knowledge. This statement is not supported by the evidence in our study. In fact, in postsurgical patients, responses have been found to advocate seeking of medical care [16].

Documenting how people learn about their condition through personal Facebook pages provides evidence to tailor interventions and information for people who use social media as a health-related resource for patients and caregivers to improve psychosocial and clinical outcomes. Clinicians and hospitals may encourage and facilitate social media participation. In a social media group, there is a lot of disease-related information exchange, which may increase access to health information and reduce information asymmetry between health care professionals and patients [17]. In a study about social support before and after bariatric surgery, they found that web-based social support groups enhance weight loss in this particular group [18]. Hence, together, medicine and web-based social support groups improve health care [18]. However, if clinicians do not monitor the accuracy and appropriateness of exchanged information, there is a risk of misinformation. Therefore, health care systems should educate patients and caregivers on how to evaluate information acquired through social media [19].

### Limitations

There are some limitations to this study, which must be taken into consideration. When scraping the Facebook messages, various data were lost. Images and emoticons could not be automatically loaded in Excel (Microsoft Corp). Messages that only consisted of an image were not coded, while the image could consist of social support or had other meaningful content. Emoticons were displayed as unreadable characters, but they could fall under the emotional support subcategory of *virtual affection*. Nevertheless, they were coded as “no social support present.” Because these posts are not manually abstracted, there is no complete data set, and there is a possibility that we are missing important social support. To gain more insight, we conducted a sensitivity analysis with 180 (5.2%) posts. Our results show that 66% of replies and 58% of posts do not have any emoticon. In the remaining posts, most of the emoticons used were the 4-leaf clover or the rose, and in 88% of the cases, they were paired with the word “success.” This implies that the meaning of the post does not change with an emoticon and is captured the right way. These outcomes are in line with the literature, stating that most of the emoticons used in web-based communication serve verbal utterances; for example, as emotion intensity enhancers [20,21].

### Conclusions

In this study, we observed that the main purpose of women in the EPN Facebook group was to provide and receive informational or emotional support. Patient support groups could well play a more significant role in several stages of disease management and recovery. Ideally, prospective follow-up studies have to reveal to what extent such groups may influence quality of life and overall health care costs.

## Abbreviations

EPN: Essure problemen Nederland
SSBC: Social Support Behavior Code

## References

[1] Cline RJW, Frey L, Gouran D, Poole S (1999). Communication in social support groups. Handbook of Small Group Communication.

[2] Barak A, Boniel-Nissim M, Suler J (2008). Fostering empowerment in online support groups. Comput Hum Behav.

[3] Mo PKH, Coulson NS (2008). Exploring the communication of social support within virtual communities: a content analysis of messages posted to an online HIV/AIDS support group. Cyberpsychol Behav.

[4] Mohd Roffeei Siti Hajar, Abdullah N, Basar SKR (2015). Seeking social support on Facebook for children with autism spectrum disorders (ASDs). Int J Med Inform.

[5] (2022). Essure Permanent Birth Control. US Food and Drug Administration.

[6] Cutrona Ce, Suhr Ja (2016). Controllability of stressful events and satisfaction with spouse support behaviors. Commun Res.

[7] Coulson NS, Greenwood N (2012). Families affected by childhood cancer: an analysis of the provision of social support within online support groups. Child Care Health Dev.

[8] Coulson NS, Buchanan H, Aubeeluck A (2007). Social support in cyberspace: a content analysis of communication within a Huntington's disease online support group. Patient Educ Couns.

[9] Eichhorn KC (2008). Soliciting and providing social support over the internet: an investigation of online eating disorder support groups. J Comput-Mediat Commun.

[10] Evans M, Donelle L, Hume-Loveland L (2012). Social support and online postpartum depression discussion groups: a content analysis. Patient Educ Couns.

[11] Coursaris CK, Liu M (2009). An analysis of social support exchanges in online HIV/AIDS self-help groups. Comput Hum Behav.

[12] Braithwaite DO, Waldron VR, Finn J (1999). Communication of social support in computer-mediated groups for people with disabilities. Health Commun.

[13] Oh S, Syn SY (2015). Motivations for sharing information and social support in social media: a comparative analysis of Facebook, Twitter, Delicious, YouTube, and Flickr. J Assn Inf Sci Tec.

[14] Hodges SD, Kiel KJ, Kramer ADI, Veach D, Villanueva BR (2010). Giving birth to empathy: the effects of similar experience on empathic accuracy, empathic concern, and perceived empathy. Pers Soc Psychol Bull.

[15] Nambisan P (2011). Information seeking and social support in online health communities: impact on patients' perceived empathy. J Am Med Inform Assoc.

[16] Dave A, Yi J, Boothe A, Brashear H, Byrne J, Gad Y (2019). Listening to the HysterSisters: a retrospective keyword frequency analysis of conversations about hysterectomy recovery. JMIR Perioper Med.

[17] Griffiths F, Cave J, Boardman F, Ren J, Pawlikowska T, Ball R, Clarke A, Cohen A (2012). Social networks--the future for health care delivery. Soc Sci Med.

[18] Atwood ME, Friedman A, Meisner BA, Cassin SE (2018). The exchange of social support on online bariatric surgery discussion forums: a mixed-methods content analysis. Health Commun.

[19] Gage-Bouchard EA, LaValley S, Mollica M, Beaupin LK (2017). Communication and exchange of specialized health-related support among people with experiential similarity on Facebook. Health Commun.

[20] Li L, Yang Y (2018). Pragmatic functions of emoji in internet-based communication---a corpus-based study. Asian J Second Foreign Lang Educ.

[21] Yus F (2014). Not all emoticons are created equal. Ling (dis)curso.

